# ATM function and its relationship with *ATM* gene mutations in chronic lymphocytic leukemia with the recurrent deletion (11q22.3-23.2)

**DOI:** 10.1038/bcj.2016.69

**Published:** 2016-09-02

**Authors:** Y Jiang, H-C Chen, X Su, P A Thompson, X Liu, K-A Do, W Wierda, M J Keating, W Plunkett

**Affiliations:** 1 Department of Experimental Therapeutics, The University of Texas MD Anderson Cancer Center, Houston, TX, USA; 2 Department of Biostatistics, Houston, TX, USA; 3 Department of Bioinformatics and Computational Biology, Houston, TX, USA; 4 Department of Leukemia, The University of Texas MD Anderson Cancer Center, Houston, TX, USA

## Abstract

Approximately 10–20% of chronic lymphocytic leukemia (CLL) patients exhibit del(11q22–23) before treatment, this cohort increases to over 40% upon progression following chemoimmunotherapy. The coding sequence of the DNA damage response gene, ataxia-telangiectasia-mutated (*ATM*), is contained in this deletion. The residual *ATM* allele is frequently mutated, suggesting a relationship between gene function and clinical response. To investigate this possibility, we sought to develop and validate an assay for the function of ATM protein in these patients. SMC1 (structural maintenance of chromosomes 1) and KAP1 (KRAB-associated protein 1) were found to be unique substrates of ATM kinase by immunoblot detection following ionizing radiation. Using a pool of eight fluorescence *in situ* hybridization-negative CLL samples as a standard, the phosphorylation of SMC1 and KAP1 from 46 del (11q22–23) samples was analyzed using normal mixture model-based clustering. This identified 13 samples (28%) that were deficient in ATM function. Targeted sequencing of the *ATM* gene of these samples, with reference to genomic DNA, revealed 12 somatic mutations and 15 germline mutations in these samples. No strong correlation was observed between *ATM* mutation and function. Therefore, mutation status may not be taken as an indicator of ATM function. Rather, a direct assay of the kinase activity should be used in the development of therapies.

## Introduction

Recurrent cytogenetic abnormalities occur frequently in chronic lymphocytic leukemia (CLL), ~70–80% of cases exhibit recurrent chromosomal abnormalities that can be identified by fluorescence *in situ* hybridization (FISH). The most common genomic aberrations, which include deletion at 13q, 11q, 17p and 6q, as well as a trisomy of chromosome 12, were assigned prognostic values.^1^ Approximately 10–20% of previously untreated patients with CLL exhibit a substantial deletion in the q arm of chromosome 11, the site of the ataxia-telangiectasia-mutated (*ATM*) gene at (11q22.3), which is within the minimally deleted region.^2, 3^ The ATM protein is an important regulator of the DNA damage response pathway; it is therefore notable that deletion of 11q has been associated with resistance to DNA-damaging chemotherapy.^4^ Despite the strong prognostic significance of FISH analysis for 11q deletion, mutations in *ATM* may also provide clinically vital information. Regarding 11q22–23 deletion (hereafter referred to as 11q deletion), ~30–40% of such cases have been reported to have a mutation in the remaining *ATM* allele.^5^ Although neither 11q deletion nor *ATM* mutation leads to full loss of p53 function and complete chemoresistance,^5, 6^ the combination of 11q deletion and *ATM* mutation is significantly associated with impaired responses to alkylating agents and purine analogs.^7^ However, because the pathogenic potential of most missense mutations in the *ATM* gene are not known, sequencing alone may not be a reliable predictor of ATM function. Therefore, assays of ATM function might complement clinically relevant information in addition to that from FISH and *ATM* mutation analysis.

Several different approaches have been applied to assess ATM protein function in CLL cells. For instance, the functionality of ATM has been assessed through reverse transcription-PCR tests of the upregulation of a selected number of *ATM/p53* target genes in response to DNA damage.^8^ In addition, measuring damage-induced apoptosis *in vitro* by cytotoxicity assays has also provided information regarding ATM function. For example, sensitive *ATM* mutant and resistant *TP53* mutant tumors responded differently to etoposide in the presence of MDM2 inhibitors (nutlins).^9^ Furthermore, the cytotoxic response to doxorubicin is capable of distinguishing between *ATM* mutant and *ATM* wild-type tumors.^10^ These assays are limited by their reliance on end points that are not direct *ATM* substrates. A more direct approach involves the measurement of the phosphorylation of proteins that are specific substrates of the ATM kinase such as autophosphorylation of ATM or the phosphorylation of SMC1 (structural maintenance of chromosomes 1), Nbs1 and p53 following treatment with ionizing radiation (IR).^5, 6^ However, because of the complexity in the double-strand break (DSB) response, not all *ATM* substrates exert the same degree of dependence upon ATM.

In the present investigation, we sought to integrate prior efforts to establish a biomarker for ATM function in CLL cells exhibiting deletion 11q by validating an assay of ATM activity and seeking associations with mutations in the *ATM* gene determined by next-generation sequencing.

## Materials and methods

### Cell lines

GM16666 and GM16667, obtained from the Coriell Cell Repository (Coriell Institute of Medical Research, Camden, NJ, USA) and cultured in Dulbecco's modified Eagle's medium with high glucose and 20% fetal bovine serum, are matched lines derived from the AT22IJE-T A–T cell line, a fibroblast cell line originated from an ataxia-telangiectasia patient in which ATM protein is undetectable owing to a homozygous frameshift mutation at codon of 762 in the *ATM* gene.^11^ It was transfected with either an ATM expression construct (GM16667) or an empty vector (GM16666) and maintained under hygromycin selection to generate A–T-corrected and A–T-deficient stable cell lines.^12^

### CLL samples

Peripheral blood samples from 54 patients with CLL were used in this study. They were collected from patients who consented to the Declaration of Helsinki guidelines through the institutional review board–approved protocols. Peripheral blood mononuclear cells were isolated from leukemic-phase blood as described previously,^13^ and the cells were maintained at 10^7^ cells per ml concentration at 37 °C overnight or 1 h before 10 Gy irradiation with Gamma Cell 1000 irradiator (Best Theratronics, Ottawa, ON, Canada). The characteristics of 46 CLL samples in which 11q deletion was identified by fluorescent *in situ* hybridization are described in the Supplementary Table 1.

### Chemicals and antibodies

Inhibitors of DNA-PK ((DNA-dependent protein kinase; NU-7441; Calbiochem, currently Millipore, Billerica, MA, USA), ATM (KU60019; AstraZeneca, Wilmington, DE, USA) and ATR (VE-821; Selleck Chemicals, Houston, TX, USA) was stored in dimethyl sulfoxide at −80 °C. The DSB-inducing agents neocarzinostatin, etoposide and doxorubicin were all from Sigma-Aldrich (St Louis, MO, USA).

Source of antibodies are as follows: ATM phospho-Ser1981 and ATM (Millipore), SMC1 and SMC1 phospho-Ser966 (Abcam, Cambridge, MA, USA), KAP1 (KRAB-associated protein 1; BD Biosciences, San Jose, CA, USA), KAP1 phospho-Ser824 (Bethyl Laboratories, Montgomery, TX, USA) and β-actin (Sigma-Aldrich). IR DYE 680RD-conjugated goat anti-mouse immunoglobulin G and IR DYE 800CW-conjugated goat anti-rabbit immunoglobulin G (LI-COR, Lincoln, NE, USA).

### Immunoblotting

Cellular lysates were prepared and immunoblot analysis and visualization using a LI-COR Odyssey Infrared Imager (Lincoln, NE, USA) was performed as described previously.^13^ Quantitation analysis was carried out by Image Studio software (LI-COR).

### Statistical analysis

The proportions of pSMC1 to SMC and pKAP1 to KAP were taken into account when determining the function of ATM. The normal mixture model-based clustering method was used to identify groups of samples that lost ATM function in this study^14^ and details were described in the Supplementary Methods.

### Targeted sequencing of the *ATM* gene

T cells from frozen CLL patient samples were isolated and expanded using Dynabeads Human T Expander CD3/28 according to the manufacturer's instruction (Life Technology, Grand Island, NY, USA). Genomic DNAs from T cells and CLL cells were obtained using a QIAamp DNA Extraction Kit (Qiagen, Valencia, CA, USA). Primers were designed to cover all 66 exons to selectively capture and amplify the *ATM* genomic DNA sequence (SeqCap EZ Choice Library; Roche NimbleGen, Madison, WI, USA). The sequencing was performed using the Illumina Hi-Seq sequencing system in the UT MD Anderson Cancer Center DNA Analysis Core Facility (Houston, TX, USA).

### Somatic mutation detection from captured DNA sequencing

The raw paired-end reads in FASTQ format were aligned to the human reference genome (hg19), using MOSAIK^15^ alignment software. The exonic regions in ATM had about 400 × mean depth of coverage. The resulting alignments were analyzed with PCR duplicate removal using the Bayesian model-based software GigaBayes/FreeBayes^16^ (details are described in the Supplementary Methods).

### Methylation analysis with pyrosequencing

Both bisulfite conversion and subsequent pyrosequencing analysis were carried out as described in Supplementary Methods at the DNA Methylation Analysis Core (MD Anderson Cancer Center).

## Results

### Identification of ATM substrates

**Figure 1:**
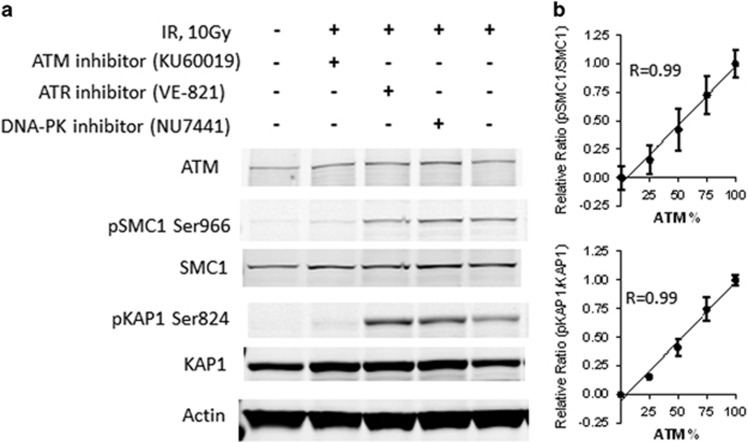
Identification of ATM substrates. (**a**) ATM-repleted (GM16667) cells were pre-treated with inhibitors of ATM, ATR and DNA-PK (KU60019, VE-821 and NU-7441, respectively) for 1 h before subjecting cells to 10 Gy of irradiation. Cell lysates were harvested 1 h after irradiation. The result is representative of two independent experiments. (**b**) ATM-deficient and -repleted cells were combined in the indicated percentages. Quantitations of pSMC1/SMC1 and pKAP1/KAP1 values were plotted against the ATM percentage. Results are based on data from four experiments.

Numerous proteins are substrates for the ATM kinase.^17^ Among them, SMC1, a member of the chromosome structure maintenance family, has been demonstrated to be an ATM-specific substrate (Ser966) in response to ionizing irradiation.^18^ KAP1 (TRIM28), a transcriptional repressor, is another ATM downstream target that is phosphorylated at Ser824 after IR treatment.^19^ Because these two sites have been reported to be specifically phosphorylated by ATM, we chose to validate them as indicators of ATM function. To this end, human fibroblasts from an A–T patient that had been complemented with *ATM* cDNA were irradiated (10 Gy), lysates were prepared 1 h later and protein phosphorylation was analyzed by immunoblotting (Figure 1a). Both KAP1 and SMC1 were phosphorylated in response to IR, but this was greatly reduced by an inhibitor of ATM kinase. In contrast, neither an inhibitor of ATR nor of DNA-PK diminished the IR-induced phosphorylation of these proteins. In addition, SMC1 and KAP1 were minimally phosphorylated in the parental ATM-null cells treated with IR (data not shown). These results indicate that SMC1 and KAP1 are ATM-specific substrates in response to IR.

### Phosphorylation of SMC1 and KAP1 is proportional to ATM protein

To further determine whether phosphorylation of SMC1 and KAP1 are linearly correlated with ATM protein level, A–T-null cells and A–T-repleted cells were mixed at the percentage proportions of 100:0, 75:25, 50:50, 25:75 and 0:100 based on cell numbers. These cell mixtures were then subjected to 10 Gy of irradiation and allowed to recover at 37 °C for 1 h before lysates were prepared. The phosphorylation levels of SMC1 and KAP1 in the mixtures were examined by immunoblots in triplicates in four independent experiments and quantitated by the Li-Cor software (Supplementary Figure 1A). As shown in Figure 1b, SMC1 and KAP1 phosphorylation increased in proportion to the amount of ATM protein. SMC1 and KAP1 phosphorylation each exhibits a linear correlation (5–75%) with the percentage of ATM amount (Supplementary Figure 1B). To determine whether the function of ATM in a cell population could be quantitated based on the phosphorylation of KAP1 and SMC1 induced by other DNA-damaging agents, we investigated the effects on SMC1 and KAP1 phosphorylation after exposure of cells to neocarzinostatin, etoposide and doxorubicin, all of which induce double-strand DNA breaks.^9, 10, 20, 21^ As described above, A–T cell mixtures were subjected to the drug treatment in place of IR, and the phosphorylation levels of SMC1 and KAP1 were quantitated to assess ATM function. However, none of these agents triggered a linear increase of SMC1 and KAP1 phosphorylation as the ATM protein level increased (Supplementary Figure 2). Based on these observations, we concluded the phosphorylation of SMC1 and KAP1 after IR was the most accurate indicator of ATM function.

### ATM function in 11q deletion CLL samples

**Figure 2:**
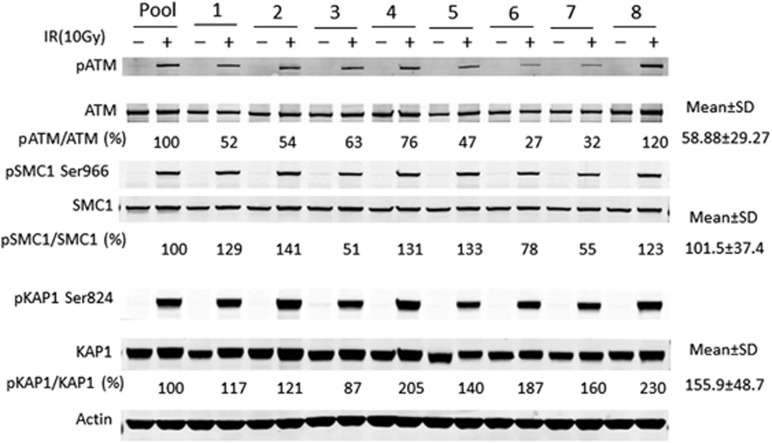
ATM function in pooled samples. Eight CLL samples from FISH normal patients were isolated and subjected to 10 Gy irradiation. The cell lysates were harvested 1 h after the treatment and immunoblotting was performed. The ratios of pATM/ATM, pSMC1/SMC1 and pKAP1/KAP1 of the Pool lysates were set as 100% and compared with those of the eight individual samples.

To establish a standard for this assay, we collected samples from eight CLL patients with normal FISH karyotypes. These samples of live cells were mock treated or irradiated (10 Gy) and protein lysates were harvested after 1 h recovery at 37 °C. The individual lysates and a Pool of equal portions of each lysate were then subjected to immunoblotting. When compared with the phospho/total values of the Pool, variations in the levels of phosphorylation of SMC1 and KAP1 were evident among the eight individual samples, indicating the heterogeneity within the CLL samples from different patients (Figure 2). We conclude that single samples are not representative of the population. Therefore, we combined lysates (Pool) for use as a positive comparator for the subsequent analyses of CLL samples that exhibit 11q deletion.

**Figure 3:**
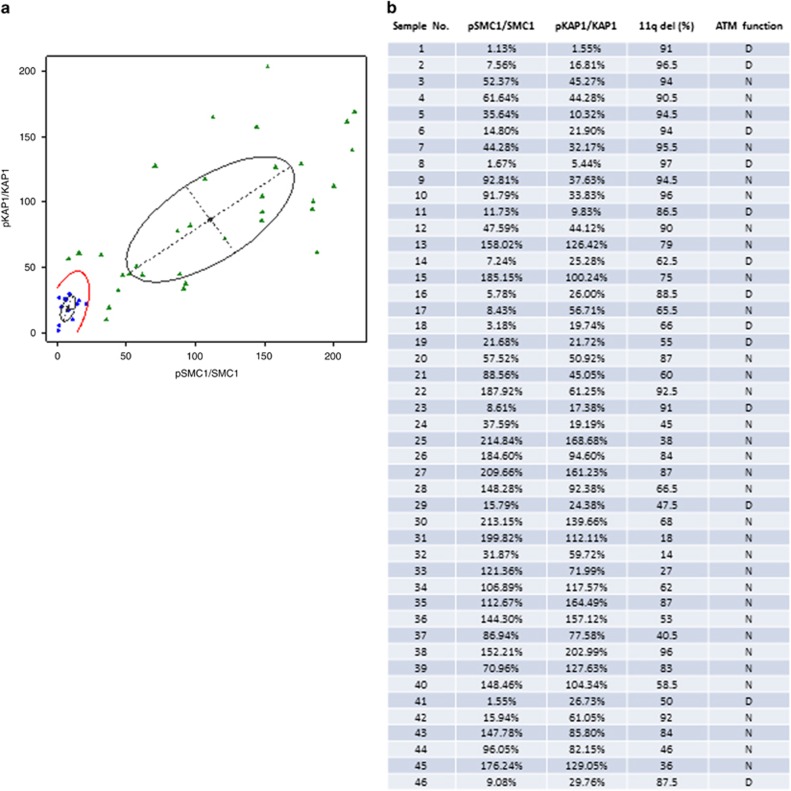
ATM function in 46 11q deletion CLL samples. (**a**) The normal mixture density and boundary for clusters estimated based for phosphorylation SMC1 and KAP1 relative the Pool. (**b**) Analysis of pSMC1/SMC1 and pKAP1/KAP1 ratios of the 46 CLL samples. Quantitation of pSMC1/SMC1 and pKAP1/KAP1 was carried out by comparing the levels in these 46 samples against the levels of those in the Pool (set as 100%) statistical formula. D, deficient; N, normal.

We applied this assay of ATM function to 46 archived or fresh CLL samples with 11q deletion to determine the function of the residual *ATM* allele. All samples were subjected to the identical treatment and evaluation as described above. The phosphorylation levels of the KAP1 and SMC1 proteins among the CLL patient samples were calculated and compared with those of the Pool comparator. The ratios of phosphorylated to total SMC1 and KAP1 from 46 CLL samples were analyzed by the normal mixture model-based clustering method.^14^ This determined that the cutoff numbers individually for both SMC1 and KAP1 to define ATM function deficiency were 21% and 55%, respectively, relative to the Pool value (Supplementary Figures 3A and B). Then, the normal mixture model-based clustering method was applied by considering the SMC1 proportion and KAP1 proportion simultaneously and a formula was generated to determine the ATM function (Figure 3a and Supplementary Figure 3C). Using this formula, we identified 13 ATM non-functional samples of 46 11q deletion CLL samples (Figure 3b). We evaluated the phosphorylation of ATM as an indicator of ATM activation, and found that the ratio of pATM to ATM did not correlate with ATM function in these CLL samples (*P*=0.49) (Supplementary Figure 4A). Although there was heterogeneity among samples of the proportion of cells that exhibited 11q deletion, the majority (37/46; 80%) of samples had the deletion in >50% of the cell population. There was no definitive association between percentage of 11q deletion and ATM function (Supplementary Figure 4B).

### Targeted sequencing of *ATM* gene sequence in 11q deletion CLL samples

**Figure 4:**
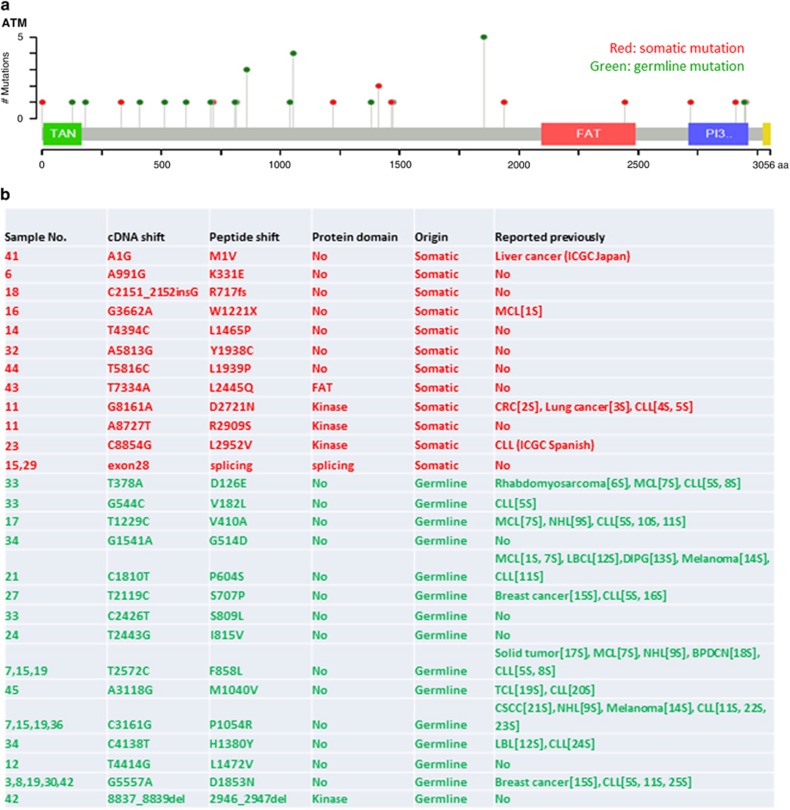
*ATM* mutations in 46 11q deletion CLL samples (>10% prevalence). (**a**) The mutation location map in the ATM protein. Green dots: germline mutations; red dots: somatic mutations. (**b**) Distribution of *ATM* mutations in 46 CLL samples. BPDCN, blastic plasmacytoid dendritic cell neoplasm; CLL, chronic lymphocytic leukemia; CRC, colon and rectal cancer; CSCC, cutaneous squamous cell carcinoma; LBCL, large B-cell lymphoma; MCL, Mantle cell lymphoma; NHL, non-Hodgkin lymphoma; TCL, T-cell leukemia. Reference listed are in the Supplementary Materials.

**Figure 5:**
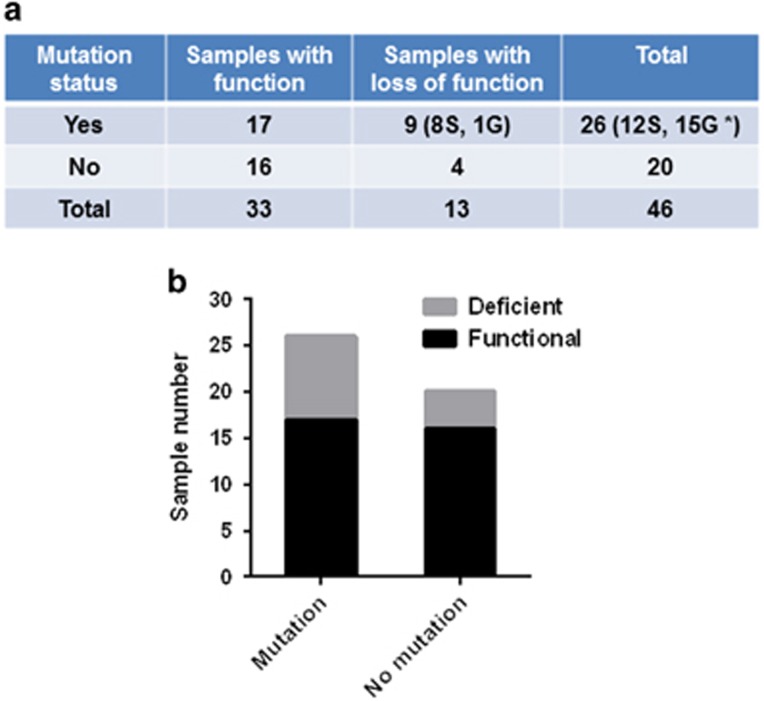
Relationship between *ATM* mutation and function. (**a**) *ATM* mutation and function distribution in all 46 CLL samples. G, germline mutation; S, somatic mutation. *One sample (no. 15) has both somatic and germline mutation. (**b**) Relationship between *ATM* mutation and function. *P*-value was calculated by Fisher's exact test.

Mutations in the residual *ATM* allele are frequently found in 11q deletion CLL samples.^5^ To determine whether there is a relationship between *ATM* gene mutation and the function of the ATM protein, we conducted targeted sequencing of the *ATM* gene in the 46 samples. Genomic DNA of T cells isolated and expanded from each of these samples was extracted to serve as a germline reference for the CLL cells, permitting comparisons of *ATM* sequence within individuals. Through a series of bioinformatics analyses, 27 *ATM* mutations were detected with a 10% variant allele frequency cutoff; the distribution of these mutations on the ATM protein is shown in Figure 4a. Among these mutations, 12 were somatic mutations. One mutation in a splicing site at exon 28 was found in two samples: a single sample (no. 11) was found to have two somatic mutations (Figure 4b). Fifteen other *ATM* mutations were recognized in this sequencing as germline mutations. In the 12 CLL samples that harbor somatic mutations, 8 were deficient in ATM function. Only one of the 15 CLL samples (7%) with only germline mutations had diminished the ATM function, indicating that the germline mutations have minimal impact on ATM activity. Seventeen of the 26 samples with mutations retained ATM function. However, 4 of the 20 CLL samples in which no mutations were detected by targeted sequencing were determined to be non-functional (Figure 5a). Based on the sequencing results, no strong correlation was observed between the presence or absence of mutations in the residual *ATM* gene of CLL cells with deletion 11q and ATM function (*P*=0.34) (Figure 5b).

In considering a cause for the lack of function in the four samples that lacked mutations, we observed that all four possessed <7% of Pool values of ATM protein levels (Table 1). Analysis of methylation patterns of the *ATM* gene was conducted to determine whether epigenetic silencing could account for low expression of ATM in these samples (Table 1). However, no evidence of excessive methylation was found in the ATM promoter region or intron–exon boundary for any of the samples. RNA was not available from these samples to quantitate ATM transcript expression analysis by reverse transcription-polymerase chain reaction or for quantitation of noncoding RNA that could affect ATM expression.^22^ Accordingly, no mechanism was established for the diminished ATM protein in samples that did not have mutations.

The progression-free survival was examined in 41 of the 11q deletion samples in which data were available. Patients with deficient ATM function had lesser progression-free survival (23 months) as compared with those with proficient ATM function (44 months) (*P*=0.48; Supplementary Figure 4C), which is consistent with a previous report.^5^ Interestingly, the median progression-free survival (44 months) for *ATM*-mutated samples was greater than *ATM*-unmutated samples (23 months), but the difference was not significant (*P*=0.93; Supplementary Figure 4D). Future investigations of samples from homogeneously treated patients may provide a definitive answer to effect of loss of ATM function on clinical response.

## Discussion

Current research suggests that deficiency of ATM function in malignancies may serve to guide evaluation of therapeutic strategies, enlighten disease prognosis and possibly contribute to clinical trial design. The lack of a consensus on a validated indicator of ATM function in clinical samples stimulated the present investigation. SMC1 and KAP1 were confirmed as unique substrates of ATM; their phosphorylation levels after irradiation were linearly correlated with ATM activity using A–T-mutant and -repleted cell lines. The ratios of phosphoprotein to total protein of SMC1 and KAP1 were used as indicators of ATM function in response to IR treatment. Using a Pool of eight FISH-negative CLL samples as a positive standard, this assay was applied in 46 primary CLL samples exhibiting deletion of the 11q region. Thirteen ATM function-deficient individuals were identified using a statistical model. Targeted sequencing of the entire coding region of the *ATM* gene on all 46 samples revealed 12 somatic and 15 germline mutations through bioinformatic analysis. However, this analysis failed to reveal a strong correlation between ATM function and mutation in the residual allele.

DNA methylation of the promoter region has been recognized as an alternative mechanism for gene inactivation in addition to deletions and mutations. Despite that aberrant methylation of the *ATM* promoter has been reported in various cancer types such as breast cancer,^23^ head and neck cancer,^24^ non-small-cell lung cancer^25^ and in primary gastric lymphoma,^26^ one study in CLL showed no apparent methylation was detected in *ATM* promoter region.^27^ In concordance with this study, our methylation analysis in four CLL samples, which lost ATM function in the absence of a mutation, did not detect any promoter hypermethylation. ATM protein levels in all 46 samples were examined and no evident relationship between the protein and function was found. Unfortunately, RNA was unavailable from these samples to quantitate *ATM* transcript or noncoding RNA expression levels. The possibility of noncoding RNA regulation of *ATM* expression is supported by the report that miR-18a expression attenuated cellular repair of DNA DSBs by directly suppressing *ATM* in colorectal cancer and breast cancer.^28, 29^ Another study demonstrated that miR-421 suppressed *ATM* expression by targeting the 3'-untranslated region of *ATM* transcripts, and its expression was regulated by N-Myc in neuroblastoma.^22^ Post-translational modification of ATM could also have a role in regulating ATM kinase activity. ATM undergoes autophosphorylation and becomes activated in response to DNA damage.^30^ Three phosphatases, including PP2A, PP5 and WIP1, have been implicated in the control of ATM activation.^31, 32, 33^ In addition to phosphorylation, it has been reported that TIP60, an acetyltransferase, acetylates ATM on Lys3016, and this post-translational modification is important for ATM activation.^34, 35^

ATM is a master regulator in the DNA damage repair signaling pathways.^36^ Loss of ATM activity leads to the rare autosomal recessive disorder ataxia-telangiectasia. Importantly, cancer susceptibility occurs at especially high incidence (10–15%) of lymphoproliferative disorders in childhood or early adulthood.^37^ In addition to CLL, the region on chromosome 11 containing the *ATM* gene is frequently deleted in Mantle cell lymphoma and T-cell prolymphocytic leukemia.^38^ Deletion of 11q has been recognized as a poor prognostic factor for CLL in progression-free survival and overall patient survival.^2^ Several studies have been conducted to test the clinical significance of *ATM* mutations in different cohorts of CLL patients. In one study in which the mutation status of *ATM*, regardless of cytogenetic background, in a cohort of 155 CLL tumors was examined, showed that *ATM* mutations were correlated with impaired overall and treatment-free survival.^6^ In a following study, the same group discovered that the combination of 11q deletion and *ATM* mutation was associated with shorter progression-free and overall survival after first-line therapy.^5^ Further, it was found that *ATM* heterozygous mutations did not influence CLL initiation, but affected disease progression.^39^ In contrast, biallelic inactivation of *ATM* was associated with reduced patient survival.^7^ Another independent investigation showed that ATM mutations were associated with a shorter treatment-free interval.^40^ Finally, two other groups concluded that *ATM* aberrations had minimal impact on both progression-free and overall survival and could not support a major prognostic role in CLL.^41, 42^

Owing to the lack in concordance in the functional significance of *ATM* mutation, a functional assay may be useful to interpret clinical outcomes. Identification of ATM function-deficient individuals may provide prognostic indications for these subgroups to therapies that target redundant, cooperative DNA damage response pathways such as ATR, DNA-PK and PARP1 inhibition. For example, a recent study reported ATR inhibition led to synthetic lethality in ATM-defective CLL cells.^43^ Another work showed that the PARP1 inhibitor, olaparib, selectively killed ATM-deficient lymphoid tumor cells.^44^ Interestingly, one group found that ATM-defective cells displayed strong non-oncogene addiction to DNA-PKcs (DNA-dependent protein kinase catalytic subunit), and that these cells underwent apoptosis after exposure to DNA-PKcs inhibitors.^45^

Therefore, the assay described, in which of the phosphorylation of ATM-specific targets (SMC1 and KAP1) are collectively assessed, could provide a reliable way to determine ATM function in cancer patients and guide subsequent treatment options.

## Figures and Tables

**Table 1:** *ATM* promoter methylation status in seven 11q deletion CLL samples without *ATM* mutation

*Sample no.*	*ATM promoter* *Average methylation (%)*	*ATM intron 1/alternative exon 1* *Average methylation (%)*	*ATM protein level (using Pool as 100%)*	*ATM function*
13	1.12	0.78	113	Normal
22	0.92	0.64	87	Normal
25	1.42	0.65	342	Normal
1	1.16	0.52	0.3	Deficient
2	0.95	0.96	2	Deficient
8	0.91	0.76	0.4	Deficient
46	1.23	0.68	6	Deficient
S/W (intermediate methylated)	39.53	42.13		
SssI (highly methylated)	87.57	91.70		
WGA (unmethylated)	7.72	0.33		

Abbreviations: ATM, ataxia-telangiectasia-mutated; CLL, chronic lymphocytic leukemia; Sssl, SssI-treated; S/W, equimolar mixture of SssI-treated and WGA-amplified; WGA, whole genome amplification.

## References

[bib1] Dohner H, Stilgenbauer S, Benner A, Leupolt E, Krober A, Bullinger L et al. Genomic aberrations and survival in chronic lymphocytic leukemia. N Engl J Med 2000; 343: 1910–1916. 11136261 10.1056/NEJM200012283432602

[bib2] Stankovic T, Skowronska A. The role of ATM mutations and 11q deletions in disease progression in chronic lymphocytic leukemia. Leuk Lymphoma 2014; 55: 1227–1239. 23906020 10.3109/10428194.2013.829919

[bib3] Gunn SR, Hibbard MK, Ismail SH, Lowery-Nordberg M, Mellink CH, Bahler DW et al. Atypical 11q deletions identified by array CGH may be missed by FISH panels for prognostic markers in chronic lymphocytic leukemia. Leukemia 2009; 23: 1011–1017. 19158838 10.1038/leu.2008.393

[bib4] Lucas DM, Ruppert AS, Lozanski G, Dewald GW, Lozanski A, Claus R et al. Cytogenetic prioritization with inclusion of molecular markers predicts outcome in previously untreated patients with chronic lymphocytic leukemia treated with fludarabine or fludarabine plus cyclophosphamide: a long-term follow-up study of the US intergroup phase III trial E2997. Leuk Lymphoma 2015; 56: 3031–3037. 25721902 10.3109/10428194.2015.1023800 PMC4688910

[bib5] Austen B, Skowronska A, Baker C, Powell JE, Gardiner A, Oscier D et al. Mutation status of the residual ATM allele is an important determinant of the cellular response to chemotherapy and survival in patients with chronic lymphocytic leukemia containing an 11q deletion. J Clin Oncol 2007; 25: 5448–5457. 17968022 10.1200/JCO.2007.11.2649

[bib6] Austen B, Powell JE, Alvi A, Edwards I, Hooper L, Starczynski J et al. Mutations in the ATM gene lead to impaired overall and treatment-free survival that is independent of IGVH mutation status in patients with B-CLL. Blood 2005; 106: 3175–3182. 16014569 10.1182/blood-2004-11-4516

[bib7] Skowronska A, Parker A, Ahmed G, Oldreive C, Davis Z, Richards S et al. Biallelic ATM inactivation significantly reduces survival in patients treated on the United Kingdom Leukemia Research Fund Chronic Lymphocytic Leukemia 4 trial. J Clin Oncol 2012; 30: 4524–4532. 23091097 10.1200/JCO.2011.41.0852

[bib8] te Raa GD, Malcikova J, Pospisilova S, Trbusek M, Mraz M, Garff-Tavernier ML et al. Overview of available p53 function tests in relation to TP53 and ATM gene alterations and chemoresistance in chronic lymphocytic leukemia. Leuk Lymphoma 2013; 54: 1849–1853. 23614766 10.3109/10428194.2013.796058

[bib9] Best OG, Gardiner AC, Majid A, Walewska R, Austen B, Skowronska A et al. A novel functional assay using etoposide plus nutlin-3a detects and distinguishes between ATM and TP53 mutations in CLL. Leukemia 2008; 22: 1456–1459. 18200038 10.1038/sj.leu.2405092

[bib10] Navrkalova V, Sebejova L, Zemanova J, Kminkova J, Kubesova B, Malcikova J et al. ATM mutations uniformly lead to ATM dysfunction in chronic lymphocytic leukemia: application of functional test using doxorubicin. Haematologica 2013; 98: 1124–1131. 23585524 10.3324/haematol.2012.081620 PMC3696617

[bib11] Gilad S, Khosravi R, Shkedy D, Uziel T, Ziv Y, Savitsky K et al. Predominance of null mutations in ataxia–telangiectasia. Hum Mol Genet 1996; 5: 433–439. 8845835 10.1093/hmg/5.4.433

[bib12] Ziv Y, Bar-Shira A, Pecker I, Russell P, Jorgensen TJ, Tsarfati I et al. Recombinant ATM protein complements the cellular A–T phenotype. Oncogene 1997; 15: 159–167. 9244351 10.1038/sj.onc.1201319

[bib13] Chen LS, Redkar S, Bearss D, Wierda WG, Gandhi V. Pim kinase inhibitor, SGI-1776, induces apoptosis in chronic lymphocytic leukemia cells. Blood 2009; 114: 4150–4157. 19734450 10.1182/blood-2009-03-212852 PMC2774551

[bib14] Fraley C, Raftery AE, Murphy TB, Scrucca L. *mclust Version 4 for R: Normal Mixture Modeling for Model-Based Clustering, Classification, and Density Estimation*, 2002. Technical Report no. 597.

[bib15] Lee WP, Stromberg MP, Ward A, Stewart C, Garrison EP, Marth GT. MOSAIK: a hash-based algorithm for accurate next-generation sequencing short-read mapping. PLoS One 2014; 9: e90581. 24599324 10.1371/journal.pone.0090581 PMC3944147

[bib16] Marth GT, Korf I, Yandell MD, Yeh RT, Gu Z, Zakeri H et al. A general approach to single-nucleotide polymorphism discovery. Nat Genet 1999; 23: 452–456. 10581034 10.1038/70570

[bib17] Matsuoka S, Ballif BA, Smogorzewska A, McDonald ER III, Hurov KE, Luo J et al. ATM and ATR substrate analysis reveals extensive protein networks responsive to DNA damage. Science 2007; 316: 1160–1166. 17525332 10.1126/science.1140321

[bib18] Kim ST, Xu B, Kastan MB. Involvement of the cohesin protein, Smc1, in Atm-dependent and independent responses to DNA damage. Genes Dev 2002; 16: 560–570. 11877376 10.1101/gad.970602 PMC155347

[bib19] Ziv Y, Bielopolski D, Galanty Y, Lukas C, Taya Y, Schultz DC et al. Chromatin relaxation in response to DNA double-strand breaks is modulated by a novel ATM- and KAP-1 dependent pathway. Nat Cell Biol 2006; 8: 870–876. 16862143 10.1038/ncb1446

[bib20] Guo K, Shelat AA, Guy RK, Kastan MB. Development of a cell-based, high-throughput screening assay for ATM kinase inhibitors. J Biomol Screen 2014; 19: 538–546. 24464432 10.1177/1087057113520325

[bib21] Shabbeer S, Omer D, Berneman D, Weitzman O, Alpaugh A, Pietraszkiewicz A et al. BRCA1 targets G2/M cell cycle proteins for ubiquitination and proteasomal degradation. Oncogene 2013; 32: 5005–5016. 23246971 10.1038/onc.2012.522 PMC3796024

[bib22] Hu H, Du L, Nagabayashi G, Seeger RC, Gatti RA. ATM is down-regulated by N-Myc-regulated microRNA-421. Proc Natl Acad Sci USA 2010; 107: 1506–1511. 20080624 10.1073/pnas.0907763107 PMC2824372

[bib23] Vo QN, Kim WJ, Cvitanovic L, Boudreau DA, Ginzinger DG, Brown KD. The ATM gene is a target for epigenetic silencing in locally advanced breast cancer. Oncogene 2004; 23: 9432–9437. 15516988 10.1038/sj.onc.1208092

[bib24] Ai L, Vo QN, Zuo C, Li L, Ling W, Suen JY et al. Ataxia-telangiectasia-mutated (ATM) gene in head and neck squamous cell carcinoma: promoter hypermethylation with clinical correlation in 100 cases. Cancer Epidemiol Biomarkers Prev 2004; 13: 150–156. 14744748 10.1158/1055-9965.epi-082-3

[bib25] Safar AM, Spencer H III, Su X, Coffey M, Cooney CA, Ratnasinghe LD et al. Methylation profiling of archived non-small cell lung cancer: a promising prognostic system. Clin Cancer Res 2005; 11: 4400–4405. 15958624 10.1158/1078-0432.CCR-04-2378

[bib26] Huang Q, Su X, Ai L, Li M, Fan CY, Weiss LM. Promoter hypermethylation of multiple genes in gastric lymphoma. Leuk Lymphoma 2007; 48: 1988–1996. 17852707 10.1080/10428190701573224

[bib27] Mikeska T, Carney DA, Seymour JF, Dobrovic A. No evidence for DNA methylation of the ATM promoter CpG island in chronic lymphocytic leukemia. Leuk Lymphoma 2012; 53: 1420–1422. 22204453 10.3109/10428194.2011.653640

[bib28] Wu CW, Dong YJ, Liang QY, He XQ, Ng SS, Chan FK et al. MicroRNA-18a attenuates DNA damage repair through suppressing the expression of ataxia telangiectasia mutated in colorectal cancer. PLoS One 2013; 8: e57036. 23437304 10.1371/journal.pone.0057036 PMC3578802

[bib29] Song L, Lin C, Wu Z, Gong H, Zeng Y, Wu J et al. miR-18a impairs DNA damage response through downregulation of ataxia telangiectasia mutated (ATM) kinase. PLoS One 2011; 6: e25454. 21980462 10.1371/journal.pone.0025454 PMC3181320

[bib30] Bakkenist CJ, Kastan MB. DNA damage activates ATM through intermolecular autophosphorylation and dimer dissociation. Nature 2003; 421: 499–506. 12556884 10.1038/nature01368

[bib31] Goodarzi AA, Jonnalagadda JC, Douglas P, Young D, Ye R, Moorhead GB et al. Autophosphorylation of ataxia–telangiectasia mutated is regulated by protein phosphatase 2A. EMBO J 2004; 23: 4451–4461. 15510216 10.1038/sj.emboj.7600455 PMC526470

[bib32] Ali A, Zhang J, Bao S, Liu I, Otterness D, Dean NM et al. Requirement of protein phosphatase 5 in DNA-damage-induced ATM activation. Genes Dev 2004; 18: 249–254. 14871926 10.1101/gad.1176004 PMC338278

[bib33] Shreeram S, Demidov ON, Hee WK, Yamaguchi H, Onishi N, Kek C et al. Wip1 phosphatase modulates ATM-dependent signaling pathways. Mol Cell 2006; 23: 757–764. 16949371 10.1016/j.molcel.2006.07.010

[bib34] Sun Y, Jiang X, Chen S, Fernandes N, Price BD. A role for the Tip60 histone acetyltransferase in the acetylation and activation of ATM. Proc Natl Acad Sci USA 2005; 102: 13182–13187. 16141325 10.1073/pnas.0504211102 PMC1197271

[bib35] Sun Y, Xu Y, Roy K, Price BD. DNA damage-induced acetylation of lysine 3016 of ATM activates ATM kinase activity. Mol Cell Biol 2007; 27: 8502–8509. 17923702 10.1128/MCB.01382-07 PMC2169409

[bib36] Shiloh Y. ATM: expanding roles as a chief guardian of genome stability. Exp Cell Res 2014; 329: 154–161. 25218947 10.1016/j.yexcr.2014.09.002

[bib37] Taylor AM, Metcalfe JA, Thick J, Mak YF. Leukemia and lymphoma in ataxia telangiectasia. Blood 1996; 87: 423–438. 8555463

[bib38] Gumy-Pause F, Wacker P, Sappino AP. ATM gene and lymphoid malignancies. Leukemia 2004; 18: 238–242. 14628072 10.1038/sj.leu.2403221

[bib39] Skowronska A, Austen B, Powell JE, Weston V, Oscier DG, Dyer MJ et al. ATM germline heterozygosity does not play a role in chronic lymphocytic leukemia initiation but influences rapid disease progression through loss of the remaining ATM allele. Haematologica 2012; 97: 142–146. 21933854 10.3324/haematol.2011.048827 PMC3248944

[bib40] Guarini A, Marinelli M, Tavolaro S, Bellacchio E, Magliozzi M, Chiaretti S et al. ATM gene alterations in chronic lymphocytic leukemia patients induce a distinct gene expression profile and predict disease progression. Haematologica 2012; 97: 47–55. 21993670 10.3324/haematol.2011.049270 PMC3248930

[bib41] Ouillette P, Li J, Shaknovich R, Li Y, Melnick A, Shedden K et al. Incidence and clinical implications of ATM aberrations in chronic lymphocytic leukemia. Genes Chromosomes Cancer 2012; 51: 1125–1132. 22952040 10.1002/gcc.21997 PMC3465492

[bib42] Lozanski G, Ruppert AS, Heerema NA, Lozanski A, Lucas DM, Gordon A et al. Variations of the ataxia telangiectasia mutated gene in patients with chronic lymphocytic leukemia lack substantial impact on progression-free survival and overall survival: a Cancer and Leukemia Group B study. Leuk Lymphoma 2012; 53: 1743–1748. 22369572 10.3109/10428194.2012.668683 PMC3724930

[bib43] Kwok M, Davies N, Agathanggelou A, Smith E, Oldreive C, Petermann E et al. ATR inhibition induces synthetic lethality and overcomes chemoresistance in TP53- or ATM-defective chronic lymphocytic leukemia cells. Blood 2016; 127: 582–595. 26563132 10.1182/blood-2015-05-644872

[bib44] Weston VJ, Oldreive CE, Skowronska A, Oscier DG, Pratt G, Dyer MJ et al. The PARP inhibitor olaparib induces significant killing of ATM-deficient lymphoid tumor cells *in vitro* and *in vivo*. Blood 2010; 116: 4578–4587. 20739657 10.1182/blood-2010-01-265769

[bib45] Riabinska A, Daheim M, Herter-Sprie GS, Winkler J, Fritz C, Hallek M et al. Therapeutic targeting of a robust non-oncogene addiction to PRKDC in ATM-defective tumors. Sci Transl Med 2013; 5: 189ra178. 10.1126/scitranslmed.3005814 23761041

