# Supplementary material for: ATM function and its relationship with ATM gene mutations in chronic lymphocytic leukemia with the recurrent deletion (11q22.3-23.2)
Source: Blood Cancer J. 2016 Sep 2;6(9):e465–. doi: 10.1038/bcj.2016.69 (PMC5056966; doi:10.1038/bcj.2016.69)
Supplement: Supplementary Information [file bcj201669x1.docx]

**Supplementary Methods**

**Statistical analysis by normal mixture model-based clustering**

Firstly, the normal mixture model-based clustering method using the SMC1 proportion and KAP1 proportion was applied separately. Then the normal mixture model-based clustering method considering the SMC1 proportion and KAP1 proportion was applied simultaneously.

For SMC1 only, the estimated normal mixture distribution is $0.29\times N\left( 8.39,28.81 \right)+0.71\times N(114.45,3745.30).$ Two clusters are defined using the optimal cut-off of 20.9. For KAP1 only, the estimated normal mixture distribution is $0.47\times N(27.67,223.31) +0.53\times N(105.46,1998.67).$ Two clusters are defined using the optimal cut-off of 54.64. For SMC1 and KAP1 together, the estimated normal mixture distribution is $0.27\times N\left( \left( 8.04,18.90 \right),\left( \begin{matrix} 28.98 & 25.88 \\ 25.88 & 96.06 \end{matrix} \right) \right)+0.73N\left( \left( 25.88,96.06 \right),\left( \begin{matrix} 3676.92 & 1897.19 \\ 1897.19 & 2286.90 \end{matrix} \right) \right)$.

The boundary of the two clusters is

$\left\{ x: 0.27\times\phi_{1}\left( x|\mu=\left( 8.04,18.90 \right),\Sigma=\left( \begin{matrix} 28.98 & 25.88 \\ 25.88 & 96.06 \end{matrix} \right) \right)0.73\times\phi_{2}\left( x|\mu=\left( 25.88,96.06 \right),\Sigma=\left( \begin{matrix} 3676.92 & 1897.19 \\ 1897.19 & 2286.90 \end{matrix} \right) \right) \right\}$, where $\phi\left( x|\mu,\Sigma\right)=\left( 2\pi\right)^{-\frac{n}{2}}\left| \Sigma\right|^{-\frac{1}{2}}e^{-\frac{1}{2}\left( x-\mu\right)^{T}\Sigma^{-1}(x-\mu)}$.

**Somatic mutation detection from captured DNA sequencing**

The program evaluates each aligned base and its base quality value at each position to indicate putative single-nucleotide variations (SNVs) and short insertions/deletions (INDELs), and their corresponding SNV probability value (*P_SNV_*). Base quality values were converted to base probabilities corresponding to every one of the four possible nucleotides. Using a Bayesian formulation, a *P_SNV_* (or INDEL probability value, as appropriate) was calculated as the likelihood that multiple different alleles are present between the reference genome sequence and the reads aligned at that position. If the probability value exceeded a pre-specified threshold, the SNV or INDEL candidate was reported in the output. In this study, a certain *P_SNV_* cutoff value (say 0.9) was used to define a high-confidence SNV or short INDEL candidate. All known SNVs/INDELs were filtered out in UCSC dbSNP 142 (human) and 1000 human genome project SNP database. The somatic status of each SNVs (or INDELS) was determined by comparing the genotypes and its likelihood between matched normal and tumor samples.

**Methylation analysis with pyrosequencing**

Genomic DNA (1 µg) was treated with sodium bisulfite using the EZ DNA Methylation-Gold Kit (Zymo Research, Irvine, CA) according to the manufacturer’s protocol. The samples were eluted in 40 µl of M-Elution Buffer, and 2 µl (equivalent to 25 ng of bisulfite-modified DNA) were used for each PCR reaction. PCR primers for the genomic area proximal to the transcription start site (covering 8 CpG sites located -39bp to +21bp from TSS) and the intronic CpG island shore (covering 2 CpG sites located +828bp to +834bp from TSS) of the *ATM* gene were designed using the Pyromark Assay Design SW 1.0 software (Qiagen, Hilden, Germany)(Supplementary Table 2). Optimal annealing temperatures for each of these primers were tested using gradient PCR.

PCR reactions were performed in a total volume of 15 µl using ZymoTaq^TM^ DNA Polymerase (Zymo Research Corporation, Irvine, CA). PCR cycling conditions were initial denaturation 10 min at 95°C, followed by 30 sec at 95°C, 30 sec at 55°C, and 30 sec at 72°C for 50 cycles. Controls for high methylation (SssI-treated DNA), low methylation (WGA-amplified DNA), partial methylation (equimolar mixture of SssI-treated and WGA-amplified DNA) and no-DNA template were included in each reaction. Half of the volume was used for each pyrosequencing reaction as previously described[^1^](#_ENREF_1). Briefly, PCR product purification was done with streptavidin-sepharose high-performance beads (GE Healthcare Life Sciences, Piscataway, NJ), and co-denaturation of the biotinylated PCR products and sequencing primer (3.6 pmol/reaction) was conducted following the PSQ96 sample preparation guide. Sequencing was performed on a PSQ HS 96 system (Biotage AB, Uppsala, Sweden) with the PyroMark Gold Q96 CDT Reagents (Qiagen, Hilden, Germany) according to the manufacturer’s instructions. The degree of methylation was calculated using the Pyro-Q CpG 1.0.9v software (Biotage AB, Uppsala, Sweden).

**Supplementary Figures**

**Supplementary Tables**

**Supplementary Table 1. Patient characteristics**

| **Patient No.** | **Age** | **Gender** | **Rai stage** | **IgV_H_ mutation*** | **No. Prior treatment** |
| --- | --- | --- | --- | --- | --- |
| **1** | **62** | **M** | **3** | **unmut** | **2** |
| **2** | **58** | **M** | **1** | **unmut** | **1** |
| **3** | **49** | **M** | **1** | **unmut** | **0** |
| **4** | **66** | **M** | **1** | **unmut** | **0** |
| **5** | **44** | **F** | **2** | **unmut** | **0** |
| **6** | **72** | **M** | **3** | **unmut** | **0** |
| **7** | **61** | **F** | **2** | **unmut** | **0** |
| **8** | **49** | **M** | **2** | **unmut** | **0** |
| **9** | **56** | **F** | **3** | **NA** | **0** |
| **10** | **74** | **M** | **4** | **unmut** | **0** |
| **11** | **66** | **F** | **1** | **unmut** | **0** |
| **12** | **62** | **M** | **NA** | **unmut** | **2** |
| **13** | **72** | **M** | **4** | **unmut** | **2** |
| **14** | **64** | **M** | **NA** | **NA** | **0** |
| **15** | **70** | **M** | **4** | **unmut** | **3** |
| **16** | **69** | **M** | **1** | **unmut** | **0** |
| **17** | **59** | **M** | **1** | **unmut** | **1** |
| **18** | **60** | **M** | **1** | **unmut** | **0** |
| **19** | **81** | **M** | **NA** | **NA** | **0** |
| **20** | **59** | **F** | **1** | **NA** | **0** |
| **21** | **67** | **M** | **1** | **unmut** | **0** |
| **22** | **59** | **F** | **4** | **unmut** | **4** |
| **23** | **82** | **F** | **4** | **NA** | **2** |
| **24** | **57** | **F** | **1** | **NA** | **2** |
| **25** | **57** | **M** | **1** | **unmut** | **0** |
| **26** | **66** | **M** | **3** | **NA** | **2** |
| **27** | **65** | **M** | **2** | **NA** | **0** |
| **28** | **89** | **M** | **3** | **NA** | **0** |
| **29** | **62** | **M** | **1** | **unmut** | **0** |
| **30** | **75** | **M** | **1** | **NA** | **0** |
| **31** | **49** | **M** | **1** | **unmut** | **0** |
| **32** | **65** | **F** | **4** | **unmut** | **2** |
| **33** | **42** | **M** | **1** | **unmut** | **0** |
| **34** | **72** | **F** | **4** | **mut** | **3** |
| **35** | **64** | **M** | **4** | **unmut** | **5** |
| **36** | **80** | **M** | **3** | **mut** | **6** |
| **37** | **51** | **M** | **2** | **unmut** | **4** |
| **38** | **74** | **M** | **4** | **NA** | **3** |
| **39** | **66** | **M** | **3** | **unmut** | **1** |
| **40** | **67** | **M** | **4** | **NA** | **5** |
| **41** | **64** | **F** | **2** | **NA** | **1** |
| **42** | **63** | **M** | **4** | **unmut** | **2** |
| **43** | **68** | **M** | **4** | **unmut** | **4** |
| **44** | **72** | **M** | **4** | **unmut** | **3** |
| **45** | **58** | **M** | **1** | **mut** | **6** |
| **46** | **53** | **M** | **4** | **unmut** | **1** |

| **Oligo** | **Sequence** | **Product size** | **Sequence to analyze** |
| --- | --- | --- | --- |
| **ATMP-F1** | AAGAGGGTGGGTGAGAGT | 131 bp | TYGGAGTTYGAGTYGAAGGGYGAGT |
| **ATMP-Rbio1** | [Btn]CTCAAAACACTACCCCAAAACATTC |  |  |
| **ATMP-S1** | GGTGGGTGAGAGTT |  |  |
| **ATMP-Fbio2** | [Btn]TTTGGAGGGGAGGGGATGAGGA | 131 bp | CCTCTTCRCCCTCRTCRTCCTCCCCRCCCT |
| **ATMP-R2** | CCTACCCCATATCCACCAATAACCAAC |  |  |
| **ATMP-S2** | AAACTCTCACCCAC |  |  |
| **ATMI-F3** | AGATAATTTTGATTTGTGGTGAGTA | 151 bp | TYGTGAGYGTTAG |
| **ATMI-Rbio3** | [Btn]ACTAAATTTACAAAAAACCAAAATCACTC |  |  |
| **ATMI-S3** | TGATTTGTGGTGAGTAT |  |  |

**Supplementary Table 2. Pyrosequencing Methylation Analysis Primers**

**Supplementary References**

1. Estecio MR, Yan PS, Ibrahim AE, Tellez CS, Shen L, Huang TH, et al. High-throughput methylation profiling by MCA coupled to CpG island microarray. *Genome Res* 2007; **17**: 1529-1536.

2. Greiner TC, Dasgupta C, Ho VV, Weisenburger DD, Smith LM, Lynch JC, et al. Mutation and genomic deletion status of ataxia telangiectasia mutated (ATM) and p53 confer specific gene expression profiles in mantle cell lymphoma. *Proc Natl Acad Sci U S A* 2006; **103**: 2352-2357.

3. Cancer Genome Atlas N. Comprehensive molecular characterization of human colon and rectal cancer. *Nature* 2012; **487**: 330-337.

4. Suzuki A, Mimaki S, Yamane Y, Kawase A, Matsushima K, Suzuki M, et al. Identification and characterization of cancer mutations in Japanese lung adenocarcinoma without sequencing of normal tissue counterparts. *PLoS One* 2013; **8**: e73484.

5. Austen B, Skowronska A, Baker C, Powell JE, Gardiner A, Oscier D, et al. Mutation status of the residual ATM allele is an important determinant of the cellular response to chemotherapy and survival in patients with chronic lymphocytic leukemia containing an 11q deletion. *J Clin Oncol* 2007; **25**: 5448-5457.

6. Lozanski G, Ruppert AS, Heerema NA, Lozanski A, Lucas DM, Gordon A, et al. Variations of the ataxia telangiectasia mutated gene in patients with chronic lymphocytic leukemia lack substantial impact on progression-free survival and overall survival: a Cancer and Leukemia Group B study. *Leuk Lymphoma* 2012; **53**: 1743-1748.

7. Kohsaka S, Shukla N, Ameur N, Ito T, Ng CK, Wang L, et al. A recurrent neomorphic mutation in MYOD1 defines a clinically aggressive subset of embryonal rhabdomyosarcoma associated with PI3K-AKT pathway mutations. *Nat Genet* 2014; **46**: 595-600.

8. Fang NY, Greiner TC, Weisenburger DD, Chan WC, Vose JM, Smith LM, et al. Oligonucleotide microarrays demonstrate the highest frequency of ATM mutations in the mantle cell subtype of lymphoma. *Proc Natl Acad Sci U S A* 2003; **100**: 5372-5377.

9. Yuille MR, Condie A, Hudson CD, Bradshaw PS, Stone EM, Matutes E, et al. ATM mutations are rare in familial chronic lymphocytic leukemia. *Blood* 2002; **100**: 603-609.

10. Gumy-Pause F, Wacker P, Maillet P, Betts DR, Sappino AP. ATM alterations in childhood non-Hodgkin lymphoma. *Cancer Genet Cytogenet* 2006; **166**: 101-111.

11. Skowronska A, Parker A, Ahmed G, Oldreive C, Davis Z, Richards S, et al. Biallelic ATM inactivation significantly reduces survival in patients treated on the United Kingdom Leukemia Research Fund Chronic Lymphocytic Leukemia 4 trial. *J Clin Oncol* 2012; **30**: 4524-4532.

12. Athanasakis E, Melloni E, Rigolin GM, Agnoletto C, Voltan R, Vozzi D, et al. The p53 transcriptional pathway is preserved in ATMmutated and NOTCH1mutated chronic lymphocytic leukemias. *Oncotarget* 2014; **5**: 12635-12645.

13. Gronbaek K, Worm J, Ralfkiaer E, Ahrenkiel V, Hokland P, Guldberg P. ATM mutations are associated with inactivation of the ARF-TP53 tumor suppressor pathway in diffuse large B-cell lymphoma. *Blood* 2002; **100**: 1430-1437.

14. Grill J, Puget S, Andreiuolo F, Philippe C, MacConaill L, Kieran MW. Critical oncogenic mutations in newly diagnosed pediatric diffuse intrinsic pontine glioma. *Pediatr Blood Cancer* 2012; **58**: 489-491.

15. Jeck WR, Parker J, Carson CC, Shields JM, Sambade MJ, Peters EC, et al. Targeted next generation sequencing identifies clinically actionable mutations in patients with melanoma. *Pigment Cell Melanoma Res* 2014; **27**: 653-663.

16. Bozhanov SS, Angelova SG, Krasteva ME, Markov TL, Christova SL, Gavrilov IG, et al. Alterations in p53, BRCA1, ATM, PIK3CA, and HER2 genes and their effect in modifying clinicopathological characteristics and overall survival of Bulgarian patients with breast cancer. *J Cancer Res Clin Oncol* 2010; **136**: 1657-1669.

17. Britt-Compton B, Lin TT, Ahmed G, Weston V, Jones RE, Fegan C, et al. Extreme telomere erosion in ATM-mutated and 11q-deleted CLL patients is independent of disease stage. *Leukemia* 2012; **26**: 826-830.

18. Kanagal-Shamanna R, Portier BP, Singh RR, Routbort MJ, Aldape KD, Handal BA, et al. Next-generation sequencing-based multi-gene mutation profiling of solid tumors using fine needle aspiration samples: promises and challenges for routine clinical diagnostics. *Mod Pathol* 2014; **27**: 314-327.

19. Stenzinger A, Endris V, Pfarr N, Andrulis M, Johrens K, Klauschen F, et al. Targeted ultra-deep sequencing reveals recurrent and mutually exclusive mutations of cancer genes in blastic plasmacytoid dendritic cell neoplasm. *Oncotarget* 2014; **5**: 6404-6413.

20. Vorechovsky I, Luo L, Dyer MJ, Catovsky D, Amlot PL, Yaxley JC, et al. Clustering of missense mutations in the ataxia-telangiectasia gene in a sporadic T-cell leukaemia. *Nat Genet* 1997; **17**: 96-99.

21. Sutton LA, Ljungstrom V, Mansouri L, Young E, Cortese D, Navrkalova V, et al. Targeted next-generation sequencing in chronic lymphocytic leukemia: a high-throughput yet tailored approach will facilitate implementation in a clinical setting. *Haematologica* 2015; **100**: 370-376.

22. Li YY, Hanna GJ, Laga AC, Haddad RI, Lorch JH, Hammerman PS. Genomic analysis of metastatic cutaneous squamous cell carcinoma. *Clin Cancer Res* 2015; **21**: 1447-1456.

23. Stankovic T, Stewart GS, Fegan C, Biggs P, Last J, Byrd PJ, et al. Ataxia telangiectasia mutated-deficient B-cell chronic lymphocytic leukemia occurs in pregerminal center cells and results in defective damage response and unrepaired chromosome damage. *Blood* 2002; **99**: 300-309.

24. Stankovic T, Weber P, Stewart G, Bedenham T, Murray J, Byrd PJ, et al. Inactivation of ataxia telangiectasia mutated gene in B-cell chronic lymphocytic leukaemia. *Lancet* 1999; **353**: 26-29.

25. Ouillette P, Li J, Shaknovich R, Li Y, Melnick A, Shedden K, et al. Incidence and clinical implications of ATM aberrations in chronic lymphocytic leukemia. *Genes Chromosomes Cancer* 2012; **51**: 1125-1132.

26. Schaffner C, Stilgenbauer S, Rappold GA, Dohner H, Lichter P. Somatic ATM mutations indicate a pathogenic role of ATM in B-cell chronic lymphocytic leukemia. *Blood* 1999; **94**: 748-753.
